# Venoarterial extracorporeal membrane oxygenation as mechanical circulatory support in adult septic shock: a systematic review and meta-analysis with individual participant data meta-regression analysis

**DOI:** 10.1186/s13054-021-03668-5

**Published:** 2021-07-14

**Authors:** Ryan Ruiyang Ling, Kollengode Ramanathan, Wynne Hsing Poon, Chuen Seng Tan, Nicolas Brechot, Daniel Brodie, Alain Combes, Graeme MacLaren

**Affiliations:** 1 Yong Loo Lin School of Medicine, National University of Singapore, Singapore, Singapore; 2 Cardiothoracic Intensive Care Unit, National University Heart Centre, National University Hospital, Singapore, 119228 Singapore; 3 Saw Swee Hock School of Public Health, National University of Singapore, Singapore, Singapore; 4 Service de Médecine Intensive-Réanimation, Institut de Cardiologie, Assistance Publique-Hôpitaux de Paris, Hôpital Pitié-Salpêtrière, Paris, France; 5 Collège de France, Centre of Interdisciplinary Research in Biology, CNRS UMR7241, INSERM U1040, Paris, France; 6 Division of Pulmonary, Allergy, and Critical Care Medicine, Columbia University Medical Centre and New York-Presbyterian Hospital, New York, USA; 7 Sorbonne Université INSERM-UMRS 116, Institute of Cardio Metabolism and Nutrition, Paris, France

**Keywords:** ECMO, Venoarterial, Septic shock, Septic cardiomyopathy, Mechanical circulatory support

## Abstract

**Background:**

While recommended by international societal guidelines in the paediatric population, the use of venoarterial extracorporeal membrane oxygenation (VA ECMO) as mechanical circulatory support for refractory septic shock in adults is controversial. We aimed to characterise the outcomes of adults with septic shock requiring VA ECMO, and identify factors associated with survival.

**Methods:**

We searched Pubmed, Embase, Scopus and Cochrane databases from inception until 1st June 2021, and included all relevant publications reporting on > 5 adult patients requiring VA ECMO for septic shock. Study quality and certainty in evidence were assessed using the appropriate Joanna Briggs Institute checklist, and the Grading of Recommendations, Assessment, Development and Evaluations (GRADE) approach, respectively. The primary outcome was survival to hospital discharge, and secondary outcomes included intensive care unit length of stay, duration of ECMO support, complications while on ECMO, and sources of sepsis. Random-effects meta-analysis (DerSimonian and Laird) were conducted.

**Data synthesis:**

We included 14 observational studies with 468 patients in the meta-analysis. Pooled survival was 36.4% (95% confidence interval [CI]: 23.6%–50.1%). Survival among patients with left ventricular ejection fraction (LVEF) < 20% (62.0%, 95%-CI: 51.6%–72.0%) was significantly higher than those with LVEF > 35% (32.1%, 95%-CI: 8.69%–60.7%, *p* = 0.05). Survival reported in studies from Asia (19.5%, 95%-CI: 13.0%–26.8%) was notably lower than those from Europe (61.0%, 95%-CI: 48.4%–73.0%) and North America (45.5%, 95%-CI: 16.7%–75.8%). GRADE assessment indicated high certainty of evidence for pooled survival.

**Conclusions:**

When treated with VA ECMO, the majority of patients with septic shock and severe sepsis-induced myocardial depression survive. However, VA ECMO has poor outcomes in adults with septic shock without severe left ventricular depression. VA ECMO may be a viable treatment option in carefully selected adult patients with refractory septic shock.

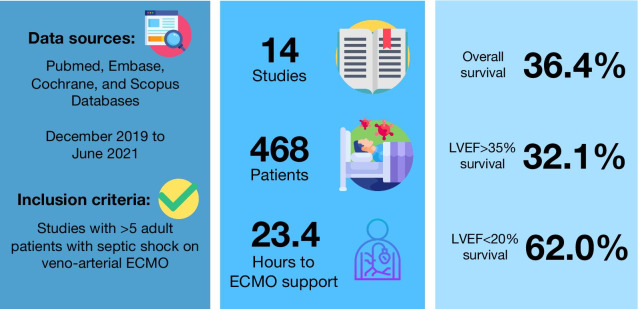

**Supplementary Information:**

The online version contains supplementary material available at 10.1186/s13054-021-03668-5.

## Background

Sepsis is a leading cause of death among critically ill patients and a global public health burden, leading to high healthcare costs [1, 2]. In 2017 alone, there were 48.9 Million cases of sepsis and 11.0 Million deaths (19.7% of all global deaths) related to sepsis [3]. The burden of sepsis is highest in early childhood, followed by a second peak in incidence in late adulthood [4]. Recognising sepsis as a global health priority, the World Health Assembly adopted a resolution to reduce its burden through better awareness, early diagnosis, and aggressive management [2]. This is reflected in national healthcare efforts, one example being the German Quality Network Sepsis which aims to decrease sepsis-related mortality [5].

Approximately, 15% of patients with sepsis develop septic shock, defined as persistent hypotension requiring vasopressors and elevated lactate levels despite adequate fluid resuscitation, where hospital mortality is in excess of 40% [3]. A subset of adult patients with septic shock develops concomitant left ventricular dysfunction, often described as septic cardiomyopathy [6]. However, septic cardiomyopathy is poorly defined in the literature and may be an underdiagnosed entity due to a lack of formal diagnostic criteria [6–10]. Adult patients with septic cardiomyopathy have 2–3 times increased mortality compared with those with septic shock alone [7]. Single-centre studies have shown dismal survival rates (10–30%) when severe left ventricular (LV) dysfunction coexisted in patients with septic shock [11].

Unlike many aetiologies of cardiomyopathy, septic cardiomyopathy is reversible, and early detection and intervention of septic cardiomyopathy in patients with septic shock may reduce mortality [11]. The encouraging outcomes of extracorporeal membrane oxygenation (ECMO) in paediatric septic shock have led to it being recommended as a potential therapy in some societal guidelines [12–18]. However, the haemodynamic pattern of septic shock is markedly different across age groups: new-born infants typically present with pulmonary hypertension and right heart failure, young children with left heart failure, and adolescents and adults with distributive shock [16]. Given the contrast in haemodynamic status between adult and paediatric shock, the use of ECMO, and in particular venoarterial ECMO (VA ECMO), in adult septic shock remains controversial.

VA ECMO has been found to be a risk factor for mortality when compared to venovenous ECMO (VV ECMO) in patients with sepsis [19–21]. This might be due to the differing indications for ECMO in sepsis (concomitant hypoxemia and right ventricular dysfunction in VV ECMO vs. cardiomyopathy and vasoplegia in VA ECMO), potentially reflecting less severe disease for patients supported with VV ECMO. Nonetheless, single-centre observational studies have shown that a subset of septic adults (specifically those with septic cardiomyopathy) may benefit from VA ECMO for mechanical circulatory support [22–24]. We conducted a systematic review of literature on the outcomes and complications of VA ECMO as mechanical circulatory support in adult patients with septic shock.

## Methods

### Search strategy and selection criteria

This study was registered with PROSPERO (CRD42020161827), and was conducted in adherence with the Preferred Reporting Items for Systematic Reviews and Meta-analyses Statement [25]. We searched Medline, Embase, Cochrane, and Scopus databases from inception to 1st June, 2021, using the following keywords and their variations: “extracorporeal membrane oxygenation”, “extracorporeal life support”, “adult” and “septic shock” (Additional File 1). We assessed all relevant studies and their citation lists to identify articles for inclusion.

All studies written in English or with English translation, reporting on five or more adult patients (≥ 18 years) with septic shock supported with VA ECMO were included [18]. We excluded any non-human or paediatric studies, and any case reports to avoid publication bias. Many centres that have published case series and observational studies also report data to the Extracorporeal Life Support Organisation (ELSO) registry. To avoid duplication of patient data, we excluded studies utilising  the ELSO registry data. In the case of overlapping patient data across two or more studies in our primary meta-analysis, we included the larger study. Two reviewers (RRL and WHP) independently screened the articles for eligibility; any conflicts were resolved by consensus or by a third reviewer (KR).

### Data collection

Data were collected independently by two reviewers (RRL and WHP) using a prespecified data extraction form; any conflicts were resolved by consensus or by a third reviewer (KR). Data collection covered study characteristics, pre-ECMO characteristics, survival to hospital discharge, and other relevant clinical outcomes. Details on the data extraction form are summarised in Additional File 2. Individual participant data (IPD) were also collected for four studies that presented data individually for each patient.

### Risk of bias assessment

Using the Joanna Briggs Institute (JBI) checklists for case series and cohort studies (Additional File 3), two reviewers (RRL and WHP) independently assessed the eligibility of studies; any conflicts were resolved by consensus or by a third reviewer (KR). The possibility of publication bias was assessed using Egger’s test.

### Statistical analysis

Statistical analyses were performed on R3.6.1, using the *meta (v4.12-0), dmetar (v0.0.9000),* and *lme4 (v1.1-23)* packages [26–28]. For continuous variables, we pooled the means from the aggregate data presented in each study as per Wan et al. [29]. The primary outcome was survival to discharge. Secondary outcomes included ICU LOS, ECMO duration, complications during ECMO, and source of infection.

We anticipated significant interstudy heterogeneity given the varied presentation of sepsis and septic shock and general lack of guidelines for patient selection and management for ECMO. As such, random-effects meta-analyses (DerSimonian and Laird) were conducted, and 95% confidence intervals (CIs) were computed using the Clopper–Pearson method [30–32]. Survival outcomes are presented as pooled proportions and 95% CIs, while dichotomous outcomes are presented as pooled risk ratios (RR) and 95% CIs. Planned subgroup analyses were conducted with continuity correction to include studies with zero events, and include: geographical location (Asia, Europe, and North America), pre-ECMO serum lactate (above and below 5 mmol/l), LVEF (< 20%, 20% to 35%, > 35%), and cardiopulmonary resuscitation (CPR) before or during ECMO. As inter-study heterogeneity can be misleadingly large when assessed using I^2^ statistics for observational studies, we used the Grading of Recommendations, Assessments, Developments and Evaluations (GRADE) approach and the tau-squared (T^2^) statistic to assess the inter-study heterogeneity [33, 34]. A sensitivity analysis was performed for all analyses by omitting one study at a time to identify outliers or influential studies.

Summary-level meta-regression was conducted when at least six data points [35, 36] were collected to explore potential sources of heterogeneity or prognostically relevant study-level covariates. One-stage IPD meta-regression was conducted using the binomial distribution and logit link to compute adjusted and unadjusted ORs [37]. Intrastudy nesting of patients was accounted for by including a random slope term that allows the treatment effect to vary between studies. Fixed effects logistic regression was conducted when intrastudy patient correlation was found to be negligible. *p* value ≤ 0.05 was considered as statistically significant.

## Results

### Study details and demographics

Of 2748 references screened, our search yielded 87 potentially relevant studies across the four databases. Sixteen studies reporting on 534 adult patients with septic shock undergoing VA ECMO were included in our systematic review [20, 22, 38–51]. All studies were retrospective and observational in nature: there was one multi-centre propensity score matched study, Eleven single-centre retrospective cohort studies, and four single-centre retrospective case series. There were nine studies from Asia, five studies from Europe, and one study from North America. One study reported on patients from both Europe and North America. There were four studies with overlapping data; two of them were excluded from the primary meta-analysis. In total, 14 studies (468 patients) were included in our primary meta-analysis (Additional File 4). The pooled mean age (13 studies, 396 patients) was 53.2 years (95%-CI: 50.6–55.9), while the pooled prevalence of male patients (13 studies, 396 patients) was 63.0% (95%-CI: 55.5%–70.3%). Pneumonia was reported in 56.7% (95%-CI; 44.0%–69.0%) of patients as the primary diagnosis. The pooled pre-ECMO serum pH (11 studies, 337 patients) and lactate (14 studies, 407 patients) were 7.15 (95%-CI: 7.13–7.17) and 7.58 mmol/L (95%-CI: 6.05–9.12 mmol/L), respectively. Patients were predominantly cannulated peripherally (11 studies femoro-femoral, 2 studies jugulo-femoral). Cardiopulmonary resuscitation (CPR) before or during ECMO was conducted in 28.9% (95%-CI: 16.9%–42.5%) of patients (9 studies, 384 patients). The pooled time to ECMO cannulation from onset of septic shock was 23.4 h (95%-CI: 20.1–26.8). Baseline demographics and patient outcomes of the included studies are summarised in Additional Files 5 and 6.

### Primary meta-analysis

**Fig. 1:**
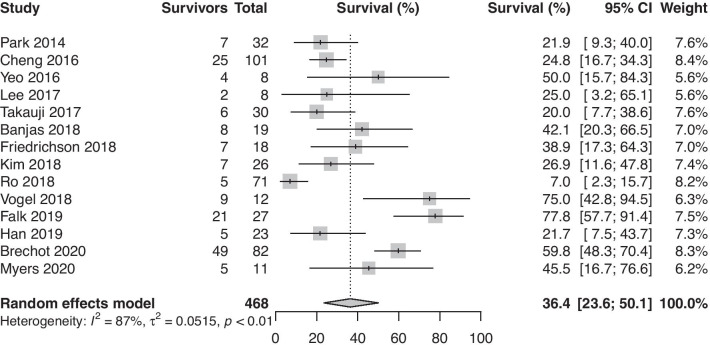
Proportion of survivors among adult patients with septic shock requiring venoarterial extracorporeal membrane oxygenation

The pooled survival to hospital discharge (14 studies, 468 patients) was 36.4% (95%-CI: 23.6%–50.1%, Fig. 1). Leave-one-out (LOO) analysis did not yield any potential outliers.

### Subgroup analysis

**Fig. 2:**
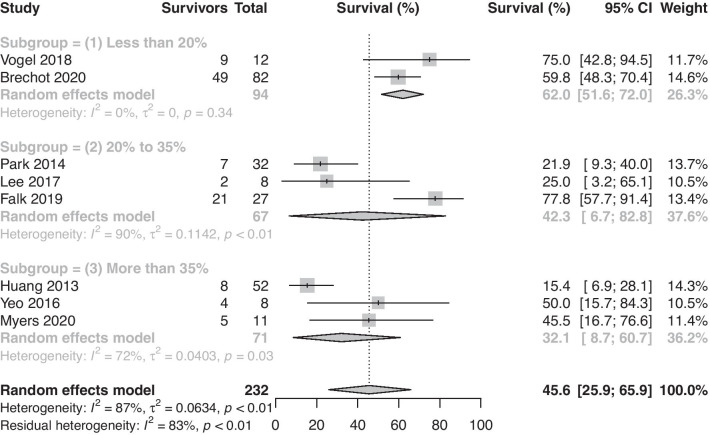
Proportion of survivors among adult patients with septic shock requiring venoarterial extracorporeal membrane oxygenation stratified by ejection fraction

Subgroup analysis yielded significant differences when considering the geographical region. Survival reported by studies from Asia (nine studies, 19.5%, 95%-CI: 13.0%–26.8%) was notably lower than in those from Europe and North America (six studies, 57.8%, 95%-CI: 44.8%–70.3%). Among five studies (190 patients), 24.4% (21 of 86) of patients undergoing CPR before or during ECMO survived, while 27.3% (54 of 144) of patients without CPR before or during ECMO survived. CPR prior to or during VA ECMO was not associated with lower survival (RR: 0.90, 95%-CI: 0.62 to 1.29, *p* = 0.55). Finally, survival reported by studies in which LVEF < 20% (three studies, (62.0%, 95%-CI: 51.6%–72.0%) was significantly higher than those where LVEF > 35% (three studies, 32.1%, 95%-CI: 8.7%–60.7%, *p* = 0.05) Survival reported by studies where LVEF was between 20 and 35% was 42.3% (95%-CI: 6.7%–82.8%, Fig. 2) Pre-ECMO serum lactate (14 studies, 407 patients) was not significantly associated with increased survival (*p* = 0.21). The results of the subgroup analysis are summarised in Table 1.

**Table 1:** Results of subgroup analysis

Subgroup		Pooled survival (%)	95% CI (%)
Geographical region (*p* < 0.001)	Asia	19.5	13.0 to 26.8
	Europe and North America	57.8	44.8 to 70.3
Presence of CPR (*p* = 0.55)	CPR	Survival = 24.4% (21 of 86)	
	No CPR	Survival = 27.3% (54 of 144)	
LVEF (*p* = 0.09)	< 20%	62.0	51.6 to 72.1
	20 to 35%	42.3	6.70 to 82.8
	> 35%	32.1	8.70 to 60.7
Serum lactate (*p* = 0.20)	< 5 mmol/l	50.5	29.8 to 71.2
	> 5 mmol/l	32.2	16.2 to 50.7

*CI* confidence interval; *VA* Venoarterial; *VV* venovenous; *CPR* cardiopulmonary resuscitation; *LVEF* left ventricular ejection fraction

### Univariable and IPD meta-regression analyses

The details of the univariable meta-regression analysis are summarised in Table 2. Age, sex, SOFA score, lactate levels, LVEF, and CPR were not associated with increased survival (Table 2). Four studies (134 patients) provided IPD on age, gender, pre-ECMO SOFA score, CPR, serum lactate, LVEF and duration of ECMO. Multivariable one-stage IPD meta-regression (Table 3) analyses revealed that age was an independent risk factor for mortality (unadjusted OR for survival: 0.974, 95% CI: 0.949–0.999, *p* = 0.04), but this association was not observed when accounting for the other covariates (adjusted OR: 0.972, 95% CI: 0.941–1.002, *p* = 0.07). Other factors were not associated with survival benefits on IPD analysis.

**Table 2:** Results of univariable meta-regression analysis

Covariate	Number of studies	Odds ratio	Lower 95% CI	Upper 95% CI	*p* value
Age	13	0.990	0.979	1.006	0.06
LVEF	8	0.991	0.978	1.002	0.13
Male sex	12	0.512	0.148	1.770	0.29
Lactate	14	0.978	0.926	1.034	0.43
SOFA	14	0.982	0.935	1.031	0.46
CPR	9	0.873	0.385	1.980	0.75
Patients with pneumonia	12	1.078	0.435	2.673	0.87
Publication year	16	1.025	0.970	1.083	0.38

*CI* confidence interval, *CPR* cardiopulmonary resuscitation; *LVEF* left ventricular ejection fraction, *SOFA* sequential organ failure assessment

**Table 3:** Results of one-stage individual patient data (IPD) meta-regression analysis

Factor	Unadjusted			Adjusted		
	OR	95% CI	*p*	OR	95% CI	*p*
ECMO duration	0.997	0.940–1.059	0.91	1.024	0.951–1.115	0.55
Lactate	0.980	0.905–1.060	0.61	0.934	0.845–1.029	0.17
CPR	0.919	0.292–3.261	0.89	0.526	0.086–3.006	0.32
SOFA score	1.030	0.929–1.136	0.55	1.028	0.878–1.211	0.74
Age	0.974	0.949–0.999	0.04	0.972	0.941–1.002	0.07
Male gender	0.609	0.293–1.248	0.18	0.742	0.309–1.769	0.46
LVEF	1.026	0.982–1.065	0.14	1.022	0.978–1.072	0.34

*OR* odds ratio; *CI* confidence interval; *ECMO* extracorporeal membrane oxygenation; *SOFA* sequential organ failure assessment; *CPR* cardiopulmonary resuscitation; *LVEF* left ventricular ejection fraction

### Secondary outcomes

The pooled ICU LOS (8 studies, 209 patients) was 19.38 days (95%-CI: 11.56–27.19). The pooled ECMO duration (10 studies, 337 patients) was 5.78 days (95%-CI: 4.11–7.45). Among 8 studies (396 patients), survivors also had significantly longer ECMO durations (+ 2.18 days, 95%-CI: 0.27–4.10, *p* = 0.03) than non-survivors. After LOO analysis, the pooled mean difference was + 2.84 days (95%-CI: 1.09–4.58, *p* = 0.002). A total of 124 complications were reported across 6 studies (198 patients). Haemorrhagic (49, 39.5%), infectious (36, 29.0%), and mechanical (23, 18.5%) were the most commonly reported complications while receiving ECMO. 9 studies (262 patients) reported on the pathogens cultured from the patients (Additional File 6). In some instances, the temporal relationship between the initiation of ECMO and positive microbiological cultures results was unclear. We could not exclude the possibility that a proportion of the cultured pathogens may have been nosocomial in origin during ECMO, rather than the inciting organism.

### Assessment of study quality

Appraisal using the JBI checklists for cohort studies and case series suggested a high level of quality across the included studies for this review, with the majority of the studies receiving at least 9/10 in the appropriate checklist (Additional File 3). Egger’s test yielded non-significant results for publication bias. A summary of the GRADE assessment for certainty of evidence is provided in Additional File 7. As the outcome was survival, the starting level of evidence for observational studies was high. Certainty for pooled survival was high, the certainty for ECMO duration was downgraded to moderate for serious imprecision, and the certainty for ICU LOS was downgraded to low for serious inconsistency and imprecision.

## Discussion

This systematic review and meta-analysis quantitatively summarised the evidence for survival of adult patients with septic shock requiring VA ECMO. Pooled survival across 14 studies and 468 patients was 36.4%. Subgroup analyses revealed that pre-ECMO LVEF significantly influenced survival rates of patients with septic shock initiated on ECMO in addition to variations in survival by geographic region of study origin.

While data are scarce, studies investigating VA ECMO adult patients with preserved LVEF have reported dismal outcomes [40, 46, 49]. It has been proposed that septic patients who have hyperdynamic left ventricular function on echocardiography have poorer outcomes than those with normo- or hypo-kinetic profiles, and this stratification may permit better patient selection for VA ECMO in septic shock [6]. A propensity-score weighted analysis found that select patients with severe myocardial dysfunction (very low LVEF) receiving VA ECMO during the first four days of septic shock had significantly lower mortality than those without ECMO [50], with similar findings among observational case series reporting on VA ECMO for adult and paediatric septic cardiomyopathy [12, 13, 47]. Concordant with these observations, our analysis found that survival among patients with LVEF > 35% was significantly lower than those with LVEF < 20% (62.0% Vs 32.1%). Patients with LVEF between 20 and 35% had intermediate survival (42.3%), suggesting a possible graded effect of LVEF on outcomes. While plausible, further research investigating pre-ECMO LVEF and its relation with mortality on VA ECMO for adult septic shock is needed to conclusively substantiate our findings.

Currently, the diagnostic criteria for adult septic cardiomyopathy are not fully established, due to the complexity and variations in the cardiovascular response to infection [52, 53]. It is also difficult to determine how well myocardial dysfunction correlates with organ dysfunction in general, and how much it independently contributes to poorer outcomes [53]. This is compounded by the lack of longitudinal echocardiography data to ascertain cardiac function at premorbid, disease, and recovery states [9]. Nonetheless, it is understood that transient and reversible myocardial depression is common in septic patients, and is associated with low or normal LV filling pressures despite depressed systolic function [54, 55]. Three broad criteria were proposed to characterise septic cardiomyopathy: LV dilatation with normal- or low-filling pressure, reduced ventricular contractility, and ventricular dysfunction with reduced response to volume infusion [8]. While increasing perfusion and cardiac output can improve survival among these patients [56, 57]. the use of very high-dose vasopressors might contribute to a vicious circle of vasoconstriction and refractory cardiovascular failure [22]. By providing mechanical circulatory support, VA ECMO can potentially restore systemic perfusion pressure and increase oxygen delivery. This corrects the cellular hypoxia and metabolic acidosis during septic cardiomyopathy, ameliorating vasopressor dependence and potentially improving the chances of survival.

In septic patients with preserved cardiac function, VA ECMO may be contraindicated as it reduces preload, and increases afterload, eventually decreasing cardiac output [58]. Of note were six patients from the study by Falk and colleagues, who underwent VV ECMO and then converted to VA ECMO. All six patients had LVEF > 35% and none of them survived to discharge. Similarly, patient profiles described by studies from Asia were characterised by distributive shock and relatively preserved LV function. On the other hand, patients in studies from Europe typically presented with severe myocardial depression, which might explain why survival reported by studies from Europe was higher than those from Asia. Apart from this, the proportion of patients undergoing CPR prior to or during ECMO, that is associated with greater mortality, was also higher in studies from Asia.

Strengths of this study include the broad inclusion criteria and relevant exclusion criteria. Our review included 14 studies, pooling data from eight different countries across three regions. We elucidated factors correlating with survival via subgroup analysis and meta-regression, reducing confounding. Coupled with non-significant results from Egger’s test, we sourced for unpublished data for IPD meta-analysis, limiting publication bias. Nonetheless, we recognise several limitations of this study. The absence of randomised studies increases the risks of confounding and bias, in particular, confounding by indication. Furthermore, there are different initiation thresholds and varying protocols and practices between individual institutions, which can introduce confounding factors given the lack of risk adjustment or propensity-scoring techniques. In addition, there was limited data on vasopressor scores or cardiac index in most of the studies. Some of the pertinent sequelae to VA ECMO such as differential oxygenation and its impact on organ dysfunction in adult septic patients could not be fully elucidated due to lack of granular data. Finally, the need for VA ECMO in adult septic cardiomyopathy is uncommon, which makes these results applicable to a narrow spectrum of patients in clinical practice. While it would be most appropriate to perform a prospective randomised clinical trial in this patient population, there would be considerable challenges in doing so, including the low incidence of patients with septic shock and septic cardiomyopathy, and the ethical challenges surrounding randomisation in ECMO studies [59–61].

## Conclusions

Our systematic review and meta-analysis of the current literature suggests that VA ECMO may be a viable salvage therapy among select patients with septic shock and concomitant myocardial depression, characterised by persistently low cardiac output refractory to inotropes. By contrast, ECMO is associated with especially poor outcomes among patients with septic shock but without severe ventricular dysfunction. Overall pooled survival in our meta-analysis was 36.4%. Patients with septic cardiomyopathy had considerably better survival than those with normal LV function. While the results of this review might only be translatable to a small population of patients with septic shock and concomitant cardiomyopathy, judicious selection of these patients for VA ECMO could improve mortality.

## Supplementary Information

**Additional file 1**. Search strings for respective databases.

**Additional file 2**. Data extraction template.

**Additional file 3**. Joanna Briggs Institute (JBI) checklists for included studies.

**Additional file 4**. Preferred Reporting Items for Systematic Review and Meta-analyses (PRISMA) flowchart for study selection.

**Additional file 5**. Baseline demographics of studies included for systematic review.

**Additional file 6**. Patient outcomes of studies included for systematic review.

**Additional file 7**. Grading of Recommendations, Assessments, Developments and Evaluations (GRADE) approach for certainty in evidence.

## Data Availability

The dataset generated and analysed during the current study can be found in the included studies and their supplementary information files.

## Abbreviations

LV: Left ventricular
ECMO: Extracorporeal membrane oxygenation
VA: Venoarterial
VV: Venovenous
LVEF: Left ventricular ejection fraction
SOFA: Sequential organ failure assessment
ICU: Intensive care unit
LOS: Length of stay
IPD: Individual participant data
JBI: Joanna Briggs Institute
RR: Risk ratio
CPR: Cardiopulmonary resuscitation
GRADE: Grading of recommendations, assessment, development, and evaluations
