# Supplementary material for: Venoarterial extracorporeal membrane oxygenation as mechanical circulatory support in adult septic shock: a systematic review and meta-analysis with individual participant data meta-regression analysis
Source: Crit Care. 2021 Jul 14;25:246. doi: 10.1186/s13054-021-03668-5 (PMC8278703; doi:10.1186/s13054-021-03668-5)
Supplement: Supplementary file 1 — Additional file 1. Search strings for respective databases. [file 13054_2021_3668_MOESM1_ESM.docx]

**Additional File 1.** Search strings for respective databases

PUBMED (MEDLINE)

| 1. | Extracorporeal Membrane Oxygenation[MeSH] OR Extracorporeal Membrane Oxygenat*[Title/Abstract] OR Extracorporeal Membrane Oxygenation[Title/Abstract] OR Extracorporeal Life Support[Title/Abstract] OR Extracorporeal Circulation[Title/Abstract] | 25,162 |
| --- | --- | --- |
| 2. | Shock, Septic[MeSH] OR Sepsis[Title/Abstract] OR Septic Shock[Title/Abstract] OR Septicemia[Title/Abstract] OR Toxic Shock Syndrome[Title/Abstract] OR pyemia[Title/Abstract] OR blood poisoning[Title/Abstract] OR septic poisoning[Title/Abstract] OR severe sepsis[Title/Abstract] | 143,362 |
| 3. | Children[Title/Abstract] OR neonate[Title/Abstract] OR adolescent[Title/Abstract] OR infant[Title/Abstract] OR pediatric[Title/Abstract] | 1,573,593 |
| 4. | #1 AND #2 NOT #3 | 715 |

COCHRANE

| 1. | MeSH descriptor: [Extracorporeal Membrane Oxygenation] explode all trees | 184 |
| --- | --- | --- |
| 2. | ((Extracorporeal) AND ((Membrane AND Oxygena*) OR (Life Support) OR Circulation OR Oxygenation)):ti,ab,kw | 2,034 |
| 3. | MeSH descriptor: [Shock, Septic] explode all trees | 1000 |
| 4. | (Sepsis OR Septic Shock OR Septicemia OR Toxic Shock Syndrome OR pyemia OR blood poisoning OR septic poisoning OR severe sepsis):ti,ab,kw | 14,321 |
| 5. | (Children OR neonate OR adolescent OR infant OR pediatric):ti,ab,kw | 265,766 |
| 8. | (#1 OR #2) AND (#3 OR #4) NOT #5 | 74 |

EMBASE

| 1. | ‘Extracorporeal oxygenation’/exp OR 'extracorporeal membrane oxygenation':ti,ab OR 'extra-corporeal membrane oxygenation':ti,ab OR 'extracoporeal membrane oxygenat*':ti,ab OR  'extracorporeal life support':ti,ab OR 'extracorporeal circulation':ti,ab | 41,788 |
| --- | --- | --- |
| 4. | ‘septic shock’/exp OR 'sepsis':ti,ab OR 'septic shock':ti,ab OR  'septicemia':ti,ab OR 'toxic shock syndrome':ti,ab OR 'pyemia':ti,ab OR 'blood poisoning':ti,ab OR 'septic poisoning':ti,ab OR 'severe sepsis':ti,ab | 221,450 |
| 7. | 'children':ti,ab OR 'neonate':ti,ab OR 'adolescent':ti,ab OR 'infant':ti,ab OR 'pediatric':ti,ab | 2,010,534 |
| 8. | #1 AND #2 NOT #3 | 1,765 |

SCOPUS

| 1. | TITLE-ABS-KEY((({extracorporeal} OR {extra-corporeal} OR {extra corporeal}) AND {membrane} AND {oxygenation}) OR {extracorporeal membrane oxygenation} OR {extra-corporeal membrane oxygenation} OR {extra corporeal membrane oxygenation} OR {ECMO} OR {extracorporeal oxygenation} OR {extra-corporeal oxygenation} OR {extra corporeal oxygenation} OR {extracorporeal life support} OR {extra-corporeal life support} OR {extra corporeal life support} OR {ECLS} OR {extrapulmonary oxygenation} OR {extracorporeal membrane oxygenation device}) | 28,234 |
| --- | --- | --- |
| 2. | TITLE-ABS-KEY ( sepsis  OR septic OR septicaemia  OR {septic shock} OR {toxic shock}  OR {Blood poisoning} OR pyohemia OR  pyemia OR {severe sepsis} ) | 257,253 |
| 3. | TITLE-ABS-KEY (child*  OR adolescent OR infant  OR neonate OR neonatal OR p*ediatric OR teen) | 5,282,446 |
| 4. | #1 AND #2 AND NOT #3 | 1,399 |
