# Supplementary material for: Venoarterial extracorporeal membrane oxygenation as mechanical circulatory support in adult septic shock: a systematic review and meta-analysis with individual participant data meta-regression analysis
Source: Crit Care. 2021 Jul 14;25:246. doi: 10.1186/s13054-021-03668-5 (PMC8278703; doi:10.1186/s13054-021-03668-5)
Supplement: Supplementary file 2 — Additional file 2. Data extraction template. [file 13054_2021_3668_MOESM2_ESM.docx]

**Additional File 2.** Data extraction template

Study characteristics: study design, study duration, year of publication, country of ECMO centre

Patient demographics: number of patients, proportion of male/female patients, mean age, pre-ECMO cardiac arrest, primary diagnosis

Pre-ECMO characteristics: shock-to-ECMO interval, pH, serum lactate, ejection fraction, SOFA score

Other relevant clinical outcomes: intensive care unit and hospital length of stay, ECMO duration, complications on ECMO.
