# Supplementary material for: Venoarterial extracorporeal membrane oxygenation as mechanical circulatory support in adult septic shock: a systematic review and meta-analysis with individual participant data meta-regression analysis
Source: Crit Care. 2021 Jul 14;25:246. doi: 10.1186/s13054-021-03668-5 (PMC8278703; doi:10.1186/s13054-021-03668-5)
Supplement: Supplementary file 3 — Additional file 3. Joanna Briggs Institute (JBI) checklists for included studies. [file 13054_2021_3668_MOESM3_ESM.docx]

**Additional File 3: Joanna Briggs Institute (JBI) checklists for included studies**

**JBI checklist for cohort studies**

| Study  (1^st^ author, year) | Question no. | | | | | | | | | | | Overall |
| --- | --- | --- | --- | --- | --- | --- | --- | --- | --- | --- | --- | --- |
|  | 1 | 2 | 3 | 4 | 5 | 6 | 7 | 8 | 9 | 10 | 11 |  |
| Park, 2014 | **✓** | **NA** | **✓** | **✓** | **✓** | **✓** | **✓** | **✓** | **✓** | **✓** | **✓** | 10/10 |
| Cheng, 2016 | **✓** | **NA** | **✓** |  | **✓** | **✓** | **✓** | **✓** | **✓** | **✓** | **✓** | 9/10 |
| Takauji, 2017 | **✓** | **NA** | **✓** | **✓** | **✓** | **✓** | **✓** | **✓** | **✓** | **✓** | **✓** | 10/10 |
| Banjas, 2018 | **✓** | **NA** | **✓** | **✓** | **✓** | **✓** | **✓** | **✓** | **✓** | **✓** | **✓** | 10/10 |
| Friedrichson, 2018 | **✓** | **NA** | **✓** | **✓** | **✓** |  | **✓** | **✓** | **✓** | **✓** | **✓** | 9/10 |
| Kim, 2018 | **✓** | **NA** | **✓** | **✓** | **✓** | **✓** | **✓** | **✓** | **✓** | **✓** | **✓** | 10/10 |
| Ro, 2018 | **✓** | **NA** | **✓** | **✓** | **✓** |  | **✓** | **✓** | **✓** | **✓** | **✓** | 9/10 |
| Falk, 2019 | **✓** | **NA** | **✓** | **✓** | **✓** |  | **✓** | **✓** | **✓** | **✓** | **✓** | 9/10 |
| Han, 2019 | **✓** | **NA** | **✓** | **✓** | **✓** |  | **✓** | **✓** | **✓** | **✓** | **✓** | 9/10 |
| Brechot, 2020 | **✓** | **NA** | **✓** | **✓** | **✓** | **✓** | **✓** | **✓** | **✓** | **✓** | **✓** | 10/10 |
| Myers, 2020 | **✓** | **NA** | **✓** | **✓** | **✓** |  | **✓** | **✓** | **✓** | **✓** | **✓** | 9/10 |

**JBI checklist for case series**

| Study  (1^st^ author, year) | Question no. | | | | | | | | | | Overall |
| --- | --- | --- | --- | --- | --- | --- | --- | --- | --- | --- | --- |
|  | 1 | 2 | 3 | 4 | 5 | 6 | 7 | 8 | 9 | 10 |  |
| Yeo, 2016 | **✓** | **?** | **?** | **?** | **?** | **✓** | **✓** |  | **✓** | **✓** | 5/10 |
| Lee, 2017 | **✓** | **✓** | **✓** | **✓** | **✓** | **✓** | **✓** | **✓** | **✓** | **✓** | 10/10 |
| Vogel, 2018 | **✓** | **✓** | **✓** | **✓** | **✓** | **✓** | **✓** | **✓** | **✓** | **✓** | 10/10 |
