# Supplementary material for: Venoarterial extracorporeal membrane oxygenation as mechanical circulatory support in adult septic shock: a systematic review and meta-analysis with individual participant data meta-regression analysis
Source: Crit Care. 2021 Jul 14;25:246. doi: 10.1186/s13054-021-03668-5 (PMC8278703; doi:10.1186/s13054-021-03668-5)
Supplement: Supplementary file 4 — Additional file 4. Preferred Reporting Items for Systematic Review and Meta-analyses (PRISMA) flowchart for study selection. [file 13054_2021_3668_MOESM4_ESM.doc]

**Additional File 4.** Preferred Reporting Items for Systematic reviews and Meta-Analyses flowchart

**Screening**

**Included**

**Eligibility**

**Identification**

Records identified through database searching
(n = 3953)

- PubMed = 715
- Embase = 1765
- Cochrane = 74
- Scopus = 1399

Additional records identified through other sources
(n = 0)

Records after duplicates removed
(n = 2748)

Records screened
(n = 2748)

Records excluded

(n = 2,661)

- Pediatric/non-human studies: 250
- Irrelevant publication type: 1257
- Not on ECMO/septic shock: 1123

Full-text articles assessed for eligibility
(n = 87)

Full-text articles excluded

(n = 71)

- Irrelevant publication type: 51
- Insufficient patients (<5): 20

Studies included in qualitative synthesis
(n = 15)

Studies included in quantitative synthesis (meta-analysis)
(n = 14)
