# Supplementary material for: Venoarterial extracorporeal membrane oxygenation as mechanical circulatory support in adult septic shock: a systematic review and meta-analysis with individual participant data meta-regression analysis
Source: Crit Care. 2021 Jul 14;25:246. doi: 10.1186/s13054-021-03668-5 (PMC8278703; doi:10.1186/s13054-021-03668-5)
Supplement: Supplementary file 5 — Additional file 5. Baseline demographics of studies included for systematic review. [file 13054_2021_3668_MOESM5_ESM.docx]

**Additional File 5.** Baseline demographics of studies included for systematic review

| Study author | Year | Sample Size | Age, Years | % male | % Pneumonia | % CPR | Pre-ECMO lactate  (mmol/l) | Pre-ECMO LVEF | Pre-ECMO pH | Cannulation method |
| --- | --- | --- | --- | --- | --- | --- | --- | --- | --- | --- |
| Huang* | 2013 | 52 | 54.37±15.94 | 75 | 48.1 | 40.4 | 8.26±5.35 | 55.7±5.78 | 7.30±1.02 | Femoro-femoral, option of central |
| Park | 2014 | 32 | 54±6.21 | 65.6 | NR | 43.8 | 9.77±6.83 | 28.7±16.3 | NR | Femoro-femoral |
| Cheng* | 2016 | 101 | NR | NR | 34.7 | 28.7 | NR | NR | NR | Femoro-femoral |
| Yeo | 2016 | 8 | 50.9±5.9 | 87.5 | 62.5 | NR | 10±5.33 | 37.9±15.5 | 7.17±0.09 | NR |
| Lee | 2017 | 8 | 47.13±14.28 | 75 | 62.5 | 75 | 12.52±4.76 | 20±16.73 | NR | Femoro-femoral |
| Takauji | 2017 | 30 | 55±24.91 | 66.7 | 26.7 | NR | 8.23±6.31 | NR | NR | NR |
| Banjas | 2018 | 19 | 63.33±14.42 | 73.7 | 52.6 | 15.8 | 3.85±4.86 | NR | 7.21±0.15 | Femoro-femoral |
| Friedrichson | 2018 | 18 | 58.25±20.31 | 72.2 | 55.6 | 11.1 | 2.67±2.21 | NR | 7.18±0.14 | Jugulo-femoral and femoro-femoral |
| Kim | 2018 | 26 | NR | NR | NR | 69.2 | NR | NR | NR | Femoro-femoral |
| Ro | 2018 | 71 | 57.33±12.11 | 56.3 | 83.3 | 12.7 | 10.8±6.58 | NR | 7.23±0.11 | Femoro-femoral |
| Vogel | 2018 | 12 | 38.08±22.01 | 41.7 | 55.6 | NR | 4.97±1.84 | 15.33±3.66 | 7.13±0.12 | Femoro-femoral |
| Falk | 2019 | 27 | 53.56±15.15 | 66.7 | NR | 25.9 | 8.44±4.21 | 31.88±16.73 | 7.11±0.24 | Jugulo-femoral |
| Han | 2019 | 23 | 51.7±27.5 | 60.8 | 78.0 | NR | 6.26±3.09 | NR | 7.15±0.24 | Femoro-femoral |
| Brechot* | 2020 | 82 | 48±15 | 43.9 | 54.5 | 13.4 | 8.9±4.4 | 17.1±7.3 | 7.13±0.15 | Femoro-femoral |
| Myers | 2020 | 11 | NR | NR | 54.5 | NR | 5.17±3.05 | 40.33±42.41 | 7.1±0.17 | NR |

Abbreviations: ECPR: extracorporeal cardiopulmonary resuscitation, ECMO: extracorporeal membrane oxygenation, LVEF: left ventricular ejection fraction, NR: not reported

*Huang 2013 reports overlapping patient data with Cheng 2016.
