# Supplementary material for: Venoarterial extracorporeal membrane oxygenation as mechanical circulatory support in adult septic shock: a systematic review and meta-analysis with individual participant data meta-regression analysis
Source: Crit Care. 2021 Jul 14;25:246. doi: 10.1186/s13054-021-03668-5 (PMC8278703; doi:10.1186/s13054-021-03668-5)
Supplement: Supplementary file 6 — Additional file 6. Patient outcomes of studies included for systematic review. [file 13054_2021_3668_MOESM6_ESM.docx]

**Additional File 6.**  Patient outcomes of studies included for systematic review

| Study author | Year | Sample Size | Survival | ICU LOS  (days) | Days on ECMO | Complications | Pathogens cultured |
| --- | --- | --- | --- | --- | --- | --- | --- |
| Huang* | 2013 | 52 | 8 (15.4%) | 5.66 ± 9.91 | 2.24 ± 3.09 | 12 mechanical, 7 haemorrhagic | 14 G+  31 G-  5 Fungi  2 Others |
| Park | 2014 | 32 | 7 (21.9%) | 13.7 ± 17.1 | 3.37 ± 2.31 | NR | NR |
| Cheng* | 2016 | 101 | 25 (24.8%) | NR | NR | NR | NR |
| Yeo | 2016 | 8 | 4 (50%) | NR | NR | NR | NR |
| Lee | 2017 | 8 | 2 (25%) | NR | 5.25 ± 3.92 | NR | 1 G+  13 G-  2 Fungi |
| Takauji | 2017 | 30 | 6 (20%) | NR | NR | 15 haemorrhagic | 6 G+  9G-  2 Fungi |
| Banjas | 2018 | 19 | 8 (42.1%) | 34 ± 38.44 | 16 ± 14.29 | NR | NR |
| Friedrichson | 2018 | 18 | 7 (38.9%) | NR | NR | NR | 7 G+  3G-  1 Fungi  1 Virus  1 Polymicrobial  7 Unknown |
| Kim | 2018 | 26 | 7 (26.9%) | NR | NR | NR | NR |
| Ro | 2018 | 71 | 5 (7.04%) | NR | 17.2 ± 2.07 | NR | 15 G+  42 G-  10 Fungi  4 others |
| Vogel | 2018 | 12 | 9 (75%) | 22.3 ± 22.64 | 10.5 + 6.29 | 1 mechanical, 1 haemorrhagic, 3 neurologic, 1 other | 8 G+  2 G-  2 Fungi |
| Falk | 2019 | 27 | 21 (77.8%) | NR | 8.78 ± 8.12 | 1 mechanical, 2 haemorrhagic, 2 pulmonary | 17 G+  9 G-  7 Virus  4 Fungi |
| Han | 2019 | 23 | 5 (21.7%) | 15.02 ± 7.39 | 6.71 ± 2 | NR | 7 G+  2 G-  1 Virus  1 Fungi |
| Brechot | 2020 | 82 | 49 (59.8%) | 30.4 ± 25 | 5.8 ± 5.6 | 6 mechanical, 17 haemorrhagic, 27 infectious, 7 metabolic | 58 G+  24 G- |
| Myers | 2020 | 11 | 5 (45.5%) | NR | 5.9 ± 9.59 | 3 mechanical, 3 haemorrhagic, 1 neurologic, 1 pulmonary, 5 cardiovascular, 5 renal, 3 infectious, 1 other | NR |

Abbreviations: ICU LOS: ICU length of stay, ECMO: extracorporeal membrane oxygenation, NR: not reported, G+: Gram-positive bacteria, G-: gram-negative bacteria

*Huang 2013 reports overlapping patient data with Cheng 2016.
