# Supplementary material for: Venoarterial extracorporeal membrane oxygenation as mechanical circulatory support in adult septic shock: a systematic review and meta-analysis with individual participant data meta-regression analysis
Source: Crit Care. 2021 Jul 14;25:246. doi: 10.1186/s13054-021-03668-5 (PMC8278703; doi:10.1186/s13054-021-03668-5)
Supplement: Supplementary file 7 — Additional file 7. Grading of Recommendations, Assessments, Developments and Evaluations (GRADE) approach for certainty in evidence. [file 13054_2021_3668_MOESM7_ESM.docx]

**Additional File 7:** Grading of Recommendations, Assessments, Developments and Evaluations (GRADE) approach for certainty in evidence

| **№ of studies** | **Certainty assessment** | | | | | | **Effect** | | | **Certainty** | **Importance** |
| --- | --- | --- | --- | --- | --- | --- | --- | --- | --- | --- | --- |
|  | **Study design** | **Risk of bias** | **Inconsistency** | **Indirectness** | **Imprecision** | **Other considerations** | **№ of events** | **№ of individuals** | **Rate (95% CI)** |  |  |
| Survival to discharge | | | | | | | | | | | |
| 14 | observational studies | not serious | not serious^a^ | not serious | not serious | none | - | 468 | 36.4%  (23.6% to 50.1%) | ⨁⨁⨁⨁ HIGH | CRITICAL |
| Duration of ECMO (days) | | | | | | | | | | | |
| 10 | observational studies | not serious | not serious^b,d^ | not serious | Serious^c^ | none | - | 337 | 5.78  (4.11 to 7.45) | ⨁⨁⨁◯ MODERATE | IMPORTANT |
| ICU length of stay (days) | | | | | | | | | | | |
| 8 | observational studies | not serious | Serious^b^ | not serious | Serious^c^ | none | - | 209 | 19.38  (11.56 to 27.19) | ⨁⨁◯◯ LOW | IMPORTANT |

#### Explanations

a. The level of heterogeneity was high (I^2^ = 86.9%, T^2^ = 0.0492) However, subgroup analysis found significant differences among patient groups.

b. There was significant heterogeneity. However, there was acceptable variability in the point estimates, and the 95% CI mostly overlapped.

c. The width of the 95% CI is wide compared to the pooled effect.

d. Analysis between survivors and non-survivors found a significant difference in the ECMO Duration, which could account for the heterogeneity.
